# Internet-Based Brief Intervention to Prevent Unhealthy Alcohol Use among Young Men: A Randomized Controlled Trial

**DOI:** 10.1371/journal.pone.0144146

**Published:** 2015-12-07

**Authors:** Nicolas Bertholet, John A. Cunningham, Mohamed Faouzi, Jacques Gaume, Gerhard Gmel, Bernard Burnand, Jean-Bernard Daeppen

**Affiliations:** 1 Alcohol Treatment Center, Department of community medicine and health, Lausanne University Hospital, Lausanne, Switzerland; 2 National Institute for Mental Health Research, Australian National University, Canberra, Australia; 3 Centre for Addiction and Mental Health, Toronto, Canada; 4 Institute of social and preventive medicine, Department of community medicine and health, Lausanne University Hospital, Lausanne, Switzerland; Aichi Cancer Center Research Institute, JAPAN

## Abstract

**Introduction:**

Alcohol use is one of the leading modifiable morbidity and mortality risk factors among young adults.

**Study Design:**

2 parallel-group randomized controlled trial with follow-up at 1 and 6 months.

**Setting/Participants:**

Internet based study in a general population sample of young men with low-risk drinking, recruited between June 2012 and February 2013.

Intervention: Internet-based brief alcohol primary prevention intervention (IBI). The IBI aims at preventing an increase in alcohol use: it consists of normative feedback, feedback on consequences, calorific value alcohol, computed blood alcohol concentration, indication that the reported alcohol use is associated with no or limited risks for health. Intervention group participants received the IBI. Control group (CG) participants completed only an assessment.

**Main Outcome Measures:**

Alcohol use (number of drinks per week), binge drinking prevalence. Analyses were conducted in 2014–2015.

**Results:**

Of 4365 men invited to participate, 1633 did so; 896 reported low-risk drinking and were randomized (IBI: n = 451; CG: n = 445). At baseline, 1 and 6 months, the mean (SD) number of drinks/week was 2.4(2.2), 2.3(2.6), 2.5(3.0) for IBI, and 2.4(2.3), 2.8(3.7), 2.7(3.9) for CG. Binge drinking, absent at baseline, was reported by 14.4% (IBI) and 19.0% (CG) at 1 month and by 13.3% (IBI) and 13.0% (CG) at 6 months. At 1 month, beneficial intervention effects were observed on the number of drinks/week (p = 0.05). No significant differences were observed at 6 months.

**Conclusion:**

We found protective short term effects of a primary prevention IBI.

**Trial Registration:**

Controlled-Trials.com ISRCTN55991918

## Introduction

Alcohol is the most often used psychoactive substance by young adults and alcohol use is one of the leading modifiable morbidity and mortality risk factors among young adults [1,2]. The burden of alcohol among young individuals is mostly explained by a binge drinking pattern (the consumption of large quantities of alcohol over short periods of time) which is highly prevalent and is known to be associated with accidents, violence (hetero and self inflicted), sexual assaults and other negative consequences [2–4]. Therefore, from a public health point of view, preventing unhealthy alcohol use among young men in the general population would be valuable.

Several public health interventions have shown potential effects. Substance use prevention programs have been tested in schools with some evidence of efficacy on alcohol use initiation [5]. Structural measures are key for primary prevention, such as limited availability, price and tax increase, [6] but could be complemented with individualized interventions. There are however major challenges related to unhealthy alcohol use prevention, such as competing economical interests in selling alcohol containing products, the willingness to decrease all types of taxes in several jurisdictions, limited interest in developing or financing preventive interventions.

Large scale individualized intervention have been made possible with new technologies. Electronic and internet based interventions represent a potentially effective option, since they allow reaching broad populations by overcoming some of the logistic constraints of face to face interventions (geographical limitations, availability and training of counselors, delivery 24 hours a day at the subjects’ convenience). It is also a relatively inexpensive procedure [7]. Internet and computer-based electronic secondary interventions has been increasing over the past years. The same delivery method can be used for primary prevention interventions. Such interventions have already been tested with some success to prevent cannabis use [8]: a computer delivered intervention in primary care was shown to be associated with a significantly lower rate of cannabis use initiation 12 months later.

If there is growing evidence for the efficacy of internet secondary prevention brief interventions for unhealthy alcohol use [7,9–12], evidence is lacking for primary prevention interventions. Since internet based brief interventions can decrease alcohol use among people with unhealthy alcohol use, one could hypothesize that the approach might be suitable for preventing the development of unhealthy use, especially binge drinking. Nevertheless, one could also hypothesize that giving information on drinking to people who are not reporting unhealthy use (personalized feedback on the absence of consequences of use for example), or comparing the drinking of people who are drinking less to the more elevated drinking of people their age and sex (normative feedback for people reporting low use of alcohol or abstinence) might not be appropriate and might lead to increases in drinking. Indeed, an iatrogenic effect of primary prevention interventions has been questioned [13]. The question is of importance since people who are drinking less are likely to be exposed to internet based interventions when those are made available to large groups of people. Even though other studies have reported the absence of iatrogenic effect of preventive interventions among people who are drinking less, additional evidence is needed [14–16].

Therefore we conducted a randomized controlled trial (RCT) examining the efficacy of an internet based prevention intervention to prevent unhealthy alcohol use (i.e. primary prevention) compared to a control (no intervention) condition among young men who did not report unhealthy alcohol use. The goal of the intervention was to prevent an increase in alcohol use, prevent the development of monthly binge drinking or the development of alcohol use consequences.

We hypothesized that, at follow up, participants receiving the intervention would report lower alcohol use, would be less likely to report monthly binge drinking and less likely to report consequences of drinking compared to controls at 1 and 6 months.

## Methods

### Study design, study sample

We implemented a parallel-group randomized controlled clinical trial with two follow-up assessments (1 and 6 months post randomization) evaluating the impact of an internet based brief primary prevention intervention on alcohol use. The study was conducted in a sample of males from the Swiss general population, geographically diverse, coming from two of the main linguistic regions of Switzerland (French and German) with different drinking practices and cultures. All participants who meet the eligibility criteria were randomized into either the intervention or a control group, with a 1:1 ratio. Participants completed a baseline assessment before randomization. The entire study, including the one-month and the 6-month assessments was done electronically.

The trial took advantage of an ongoing cohort study (Cohort study on Substance Use Risk Factors, C-SURF) that included participants when they were attending the mandatory Swiss army recruitment process, allowing thus accessing the whole male general population at an age of around 19–20 years. C-SURF recruited participants at 3 out of 6 operating recruitment centers, including the one center operating for the entire French speaking part of Switzerland. When consenting to participate in C-SURF, participants provide their email address (most C-SURF procedures are done electronically) and consent to be contacted for studies related to C-SURF. C-SURF participants were recruited from August 2010 to July 2011. From June 2012 to February 2013, they were invited to participate in the internet prevention trial (following the C-SURF recruitment calendar), irrespective of their drinking. The present study was part of a larger internet-based intervention trial that included a primary prevention intervention study (reported herein) and a secondary prevention intervention study (for those reporting unhealthy alcohol use; reported elsewhere [17]. Invitations were sent with an accompanying short text message sent on the participants’ phone, announcing the study and encouraging them to check their emails. They were presented with the opportunity to participate in an “internet study on alcohol use among young people” (Swiss study on Young People and Alcohol). Participants were offered 15CHF (the equivalent of 10£, 13€, or 17USD at the time of the study) for their participation, after completion of the 6 months follow up (gift certificate to download music online). Reminders were sent by email in case of non-response (one week and one month after the first invitation). Invitations contained a description of the study, a consent form and a personalized link to the study website. Electronic informed consent was obtained from all participants. When accessing the study website, participants had to actively confirm their willingness to take part in the study and were then given access to the baseline assessment. The baseline assessment consisted of quantity and frequency questions on alcohol use, a list of 14 alcohol related consequences and the Alcohol Use Disorders Identification Test (AUDIT) [18,19]. The baseline assessment was used to categorize people as presenting unhealthy alcohol use (defined as reporting >14 drinks per week OR at least one episode of binge drinking (6 or more drinks per occasion) per month OR AUDIT score >8) or low-risk use (i.e. absence of unhealthy use). Those presenting low-risk use were included in the present study and randomized immediately after completing the baseline assessment. Randomization was at the individual level. It was embedded in the study website, was completely automated, without experimenter involvement. After the randomization took place, participants were either presented the intervention or the control condition. The primary prevention intervention used the information collected as part of the baseline assessment.

The follow-up took place between July 2012 and October 2013.

C-SURF and the internet trial have been approved by the Ethics Committee for Clinical Research of the Canton of Vaud (C-SURF: Protocol No. 15/2007; Internet trial: Protocol No. 260/2011, approved 22.8.2011).

### Intervention

The electronic intervention included personalized feedback on alcohol use, and general information on alcohol use and its consequences. The personalized feedback consisted of: 1) Normative feedback (comparison of the participant’s alcohol consumption per week and per occasion to the consumption of individuals of the same age in the Swiss population, based on data from the Swiss Health Survey [20] with more than 12,000 participants); 2) Feedback on reported consequences (if any); 3.) Calorific value of reported consumption (if the participant reported drinking); 4.) Computed blood alcohol concentration based on maximum reported alcohol consumption, and potential consequences; 5.) Indication of the absence of unhealthy alcohol use, with indication that the reported alcohol use is associated with no or limited risks for health. Participants received the message that no change in their current use of alcohol is necessary to avoid harmful effects of alcohol use and encouragement not to increase the current alcohol use (if any). In addition participants received information on factors of vulnerability (tolerance, family history) towards the development of alcohol use disorders. Participants had the opportunity to print their personalized feedback form, and had access to a section containing general information on alcohol use and its consequences. They also later received a copy of their personalized feedback by email.

### Control

Participants in the control group completed the same baseline assessment as did members of the intervention group, but did not receive the intervention. Instead, they were presented a screen thanking them for completing the assessment.

### Measures

Alcohol consumption was collected via quantity/frequency questions (“In a given day that you use alcohol, how many drinks are you drinking?” / “On how many days in a regular week are you drinking alcohol?”). Additionally, participants completed the Alcohol Use Disorders Identification Test (AUDIT)[18,19]. The AUDIT is a 10-question measure that has been extensively studied and validated, and has been used successfully in other electronic brief interventions [7,21]. The assessed alcohol-related consequences were: was injured or injured someone else, had a hangover, missed a class or work, performed poorly at work, got into an argument or fight with friends, had unplanned sex, had unprotected sex, damaged property, had problems with the police, received medical treatment) [22]. Subjects were asked two supplemental questions about the impact of alcohol on their physical and mental health (“Over the past year, do you think your alcohol drinking had a negative impact on your physical health” / “your mental health?”). The total of assessed consequences was 12.

Questions that are time-referenced (e.g. “over the past x months…”) were adapted to accommodate each of the follow-up points (i.e. “over the past month…”, “since the last time we asked you about your drinking…”) to avoid overlap between the different observation periods.

### Outcomes

#### Primary outcomes

The primary outcomes were assessed at 1 and 6 months. Primary outcome were: 1.) weekly alcohol consumption (number of drinks per week, where one drink contained 10g of ethanol); 2.) prevalence of monthly binge drinking (defined as 6 or more drinks in one occasion).

#### Secondary outcomes

The secondary outcomes were assessed at 6 months. They were: 1) the number of reported 12 alcohol related consequences over the past 6 months; 2.) the AUDIT score, adapted for the 6 month time reference.

### Statistical analyses

Analyses were conducted in 2014–2015. An intention-to-treat analysis was used. We first performed descriptive analyses on baseline data, then investigated the occurrence of potential selection and attrition biases (Wilcoxon rank-sum tests and Pearson Chi-square tests, when appropriate). We compared different count-models using the countfit command in STATA (which compares the fit of the Poisson, negative binomial, zero-inflated Poisson and zero-inflated negative binomial models). The comparison was based on the differences between observed and average estimated probabilities for each count. The negative binomial regression model was highly preferred to the other models.

#### Primary outcomes

Intervention impact on the mean number of drinks per week was tested with a random-effects negative binomial model. The model specified subject as random effects and treatment and time as fixed effects. A treatment by time interaction was included in the model to display the effect of the intervention over time (baseline, 1-, and 6-month). Negative binomial regression model best fitted the count distribution of number of drinks per week in the sample. Intervention impact on binge drinking prevalence was tested with a logistic regression model. Since none of the participants reported binge drinking at baseline (inclusion criteria), analyses at 1 month were adjusted for age and linguistic region and analyses at 6 months were adjusted for presence of binge drinking at 1 month (first follow-up) and age and linguistic region. An *a priori* decision was made to adjust all models for age and linguistic region, based on study design and literature. Some conscripts have the possibility to report at the recruitment centers at different ages, and age is associated with drinking evolution. Also, across Swiss regions, variations in alcohol use have been observed [23].

#### Secondary outcomes

The impact of the intervention on the AUDIT score and the number of reported consequences at 6 months was analyzed using negative binomial regression models, each controlling for the respective measure at baseline, and adjusted for age and linguistic region. All analyses were done with Stata (StataCorp. 2013. Stata Statistical Software: Release 13. College Station, TX: StataCorp LP).

## Results

**Fig 1:**
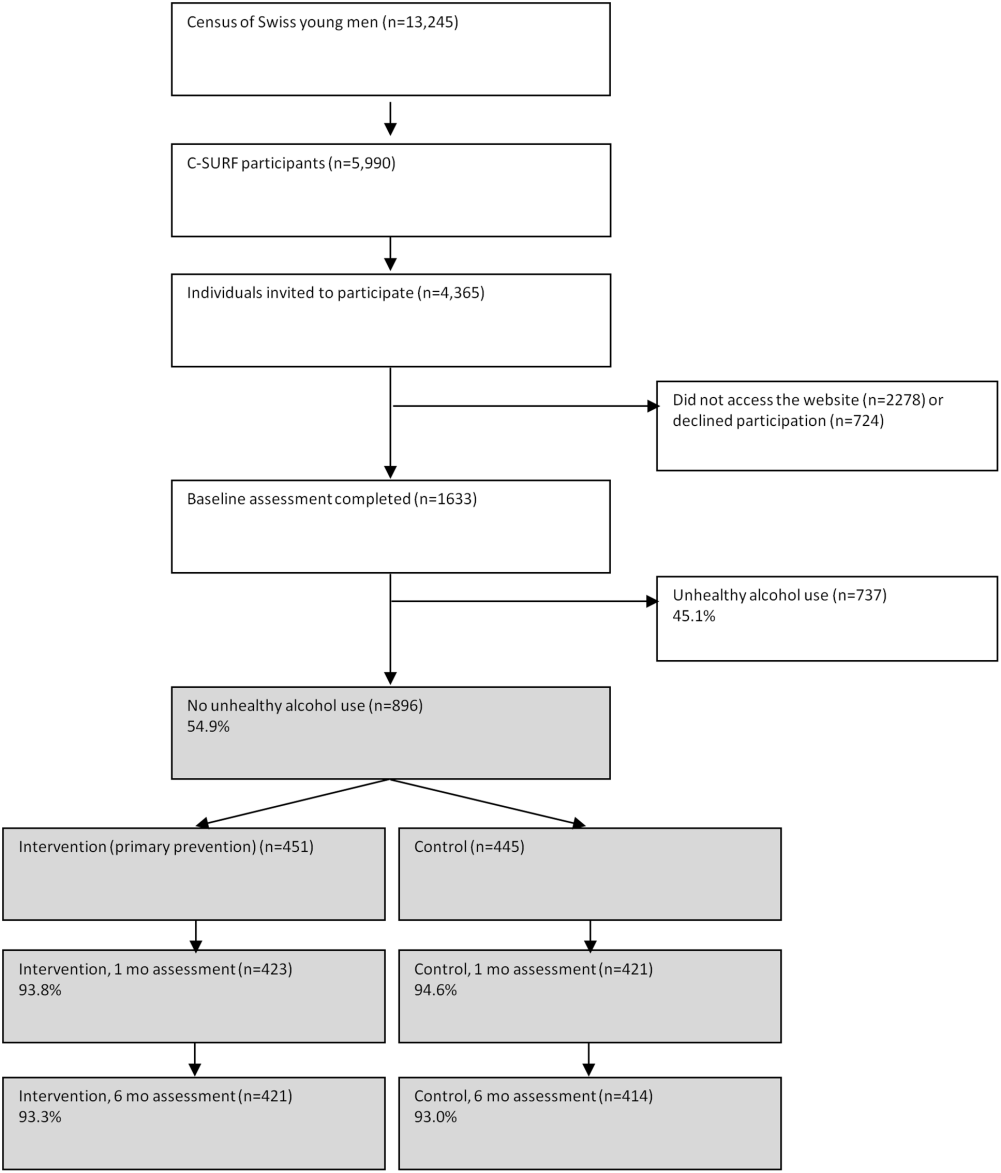
Study participant flow and follow-up rates.

Among the CSURF participants, 4365 were invited to participate in the internet study. Of those, 724 refused, 2008 never answered the invitation (after two reminders), and 1633 agreed to participate. Of those, 737 reported unhealthy alcohol use (and were included in another study), leaving a sample of 896 for the primary prevention study reported here. Participants were randomized to the intervention (n = 451) or control group (n = 445). Of the 896 participants included, 844 and 835 completed the first and second follow up, respectively (94.2% and 93.2% follow up rate). The study flow chart is presented in Fig 1.

Baseline characteristics of participants are presented in Table 1. In accordance with the inclusion criteria, no participant had unhealthy alcohol use. Therefore, none reported binge drinking. The reported mean (SD) number of drinks/week was 2.3 (2.2), the mean (SD) AUDIT score was 3.5 (2.1) and participants reported a mean (SD) of 1.0 (1.1) alcohol related consequence over the past 12 months. No differences were observed on baseline variables between participants in the intervention and control groups (all p>0.2). No differences on baseline values were observed between those with and without follow up data (all p>0.2).

**Table 1:** Baseline characteristics of participants.

	Full sample (n = 896)	Intervention group (n = 451)	Control group (n = 445)
Age, mean (SD)	21.0 (1.3)	21.0 (1.4)	20.9 (1.2)
Linguistic region:			
French speaking, n(%)	521 (58.2%)	254 (56.3%)	267 (60.0%)
German speaking, n(%)	375 (41.9%)	197 (43.7%)	178 (40.0%)
Number of drinks/week, mean(SD)	2.3 (2.2)	2.4 (2.2)	2.4 (2.3)
Binge drinking, n (%)	0 (0%)*	0 (0%)*	0 (0%)*
AUDIT score, mean (SD)	3.5 (2.1)	3.5 (2.0)	3.5 (2.1)
Number of alcohol related consequences (0–12), mean (SD)	1.0 (1.1)	1.1 (1.1)	1.0 (1.0)

* by definition (inclusion criteria)

### Number of drinks per week

At 1 month, participants in the intervention group reported a mean (SD) of 2.3 (2.6) drinks per week compared to 2.8 (3.7) in the controls. At 6 months the reported consumption was 2.5 (3.0) and 2.7 (3.9) in the intervention and control groups, respectively. The results of the regression analysis are presented in Table 2. There was a borderline intervention by time interaction at 1 month, with participants in the intervention group reporting less drinking at 1 month (IRR = 0.87 (0.76; 1.00), p = 0.05).

**Table 2:** Regression model for number of drinks per week, baseline to 6 months (n = 896).

	Number of drinks per week*, IRR (95%CI)
Treatment	
Intervention	1.04 (0.90; 1.20)
Time (reference = baseline)	
1 month	1.07 (0.98; 1.18)
6 months	1.04 (0.94; 1.14)
Treatment x Time	
1 month x intervention	0.87 (0.76; 1.00)**
6 months x intervention	0.98 (0.86; 1.13)

* Random-effects negative binomial regression model.

**p = 0.049. All models are adjusted for age and linguistic region.

OR: Odds Ratio, CI: Confidence interval, IRR: Incidence Rate Ratio.

### Binge drinking prevalence

At 1 month, 14.4% of participants in the intervention group and 19.0% of the controls reported binge drinking. Even though not statistically significant in the model adjusted for age and linguistic region (OR = 0.73 (95% CI 0.51; 1.05), p = 0.09), we would consider such difference as clinically significant. At 6 months, there were no differences between groups: 13.3% of participants in the intervention group and 13.0% of the controls reported binge drinking (OR = 1.3 (0.85; 2.04), adjusted for age, linguistic region, and presence of binge drinking at 1 month).

### AUDIT score

The mean (SD) AUDIT score at 6 months was 3.6 (2.9) and 3.7 (3.2) in the intervention and control group, respectively. No intervention effect was observed in the negative binomial regression model (IRR = 0.98 (0.89; 1.07) for the intervention group, compared to the control group at 6 months, adjusting for AUDIT score at baseline, age, and linguistic region).

### Number of consequences

At 6 months, the mean (SD) number of reported consequences was 0.7 (1.0) in the intervention group and 0.8 (1.2) in the control group. There was a protective intervention effect on the number of consequences at 6 months (IRR = 0.79(0.67; 0.94)) in negative binomial regression model adjusted for the baseline number of consequences, age and linguistic region, when comparing the intervention to the control group.

## Discussion

There is a lack of knowledge on the potential effects of primary prevention intervention for unhealthy alcohol use. Our study showed small but promising effects. We observed a statistically significant protective effect of the intervention on the number of drinks per week at 1 month but not at 6 months. We did not observe a statistically significant intervention effect on the binge drinking prevalence, nevertheless, we consider that a 6% difference in binge drinking prevalence in favor of the intervention group is notable, even though the effect did not reach a statistically significant level of 0.05. In addition, a protective effect was found on the occurrence of alcohol related consequences. This finding is at odds with electronic screening and brief intervention studies conducted among individuals with unhealthy alcohol use showing effects on drinking but not on alcohol related harm. Since the occurrence of alcohol related consequences was a secondary outcome, the impact of the intervention on consequences should be interpreted with caution.

An additional notable result is the absence of iatrogenic effects of the intervention. When internet-based interventions are made available to large populations, it may be difficult to exclude “low-risk” drinkers. Additional evidence is needed to confirm that normative feedback does not lead to an increase in drinking among people who are drinking less [14]. Our study shows that, when delivered accompanied by clear encouragements not to increase their current drinking, normative feedback did not lead to an increase in drinking. Our results are consistent with the absence of a “boomerang” effect of normative feedback showed by Prince and colleagues in four light drinking student samples [24].

Even though the observed effects were small, our results indicate potential interest for internet-based primary prevention interventions. The observed limited impact implies that, in its current form, this type of intervention cannot be seen as an alternative to other and more effective preventive measures (such as restriction to alcohol, ban on advertisement, etc.). Given the potential negative consequences of binge drinking and given its prevalence in the young population, especially among males, we think this type of intervention is of interest.

Due to the selection of people without binge drinking at baseline, we did observe an increase in binge drinking prevalence in both groups, which is consistent with a regression to the mean effect. Another explanation is that participants were assessed at the age binge drinking becomes more prevalent. In this age group in Switzerland, the prevalence of monthly binge drinking is 46% [25].

Limitations include self-assessment: because of the study design, other measures than self-assessments were not feasible and social desirability bias is possible. Another limitation is that the study was conducted in a sample consisting of males only and is therefore not indicative of what would happen in a female sample. More research is needed among young women. In addition, when we determined the study sample size, we hypothesized a larger intervention effect compared to the observed effect (due to the lack of primary prevention studies in our population we had to extrapolate from studies conducted in other populations, including in people with unhealthy alcohol use). The study was adequately powered for the hypothesized effect (80% power, 1.5 drinks per week difference). Since the observed effect was much smaller, a larger sample would have been necessary to maintain a 80% power. The study sample allowed a 63% power to detect the observed difference of 0.5 drinks per week at 1 month.

Our study showed limited effects which is in line with other studies that included primary prevention elements for substance use [14–16]. More research is needed to investigate whether or not these interventions can be modified as to lead to stronger effects.

The strength of the present study is the use of a general population sample, coming from diverse geographical and linguistic regions over two of the main Swiss linguistic regions. In addition, Switzerland has the interesting feature of comprising regions with drinking patterns similar to Nordic European countries (German speaking part) and Southern European countries (French speaking part). As such, individuals with varying drinking cultures and drinking environment were included, which is what would happen if such intervention is made available to the general population.

## Supporting Information

S1 CONSORT Checklist CONSORT checklist of information to include when reporting a randomised trial. (DOC)

S1 Protocol Protocol submitted to the Swiss National Science Foundation. (DOCX)
