# Supplementary material for: Internet-Based Brief Intervention to Prevent Unhealthy Alcohol Use among Young Men: A Randomized Controlled Trial
Source: PLoS One. 2015 Dec 7;10(12):e0144146. doi: 10.1371/journal.pone.0144146 (PMC4671673; doi:10.1371/journal.pone.0144146)
Supplement: S1 Protocol — (DOCX) [file pone.0144146.s002.docx]

Application to the Swiss National Science Foundation

**Second part: Scientific information**

Electronic screening and brief intervention (E-SBI) for young adults:

a randomized controlled trial

Nicolas Bertholet, MD, MSc

Jean-Bernard Daeppen, MD

Bernard Burnand, MD, MPH

Gerhard Gmel, PhD

International collaborator:

John Cunningham, PhD

**1. Summary of the research plan**

**Background:** Unhealthy alcohol use is a leading cause of morbidity and mortality among young adults. Face-to-face brief interventions targeting alcohol use are considered effective in primary care settings, but young individuals tend to have limited contacts with the health care system and are therefore unlikely to receive any intervention. As a result, electronic brief interventions have been developed that can reach a broad population at relatively low cost and potentially impact the public health system. Electronic screening and brief interventions (E-SBI) targeting alcohol use are effective among college students, but more evidence is needed in order to evaluate E-SBI designed for young individuals in other settings. There is also a lack of knowledge about the primary prevention effects of these interventions on, for example, moderate drinkers.

**Specific aims:** We will adapt our website [www.alcooquizz.ch](http://www.alcooquizz.ch) to evaluate the efficacy of a proactive E-SBI providing personalized feedback and information on alcohol use and its consequences among young men in the general population, and to help determine how young men will use E-SBI to obtain information on alcohol use and its consequences.

**Working hypothesis:** It is hypothesized that E-SBI will decrease later alcohol use and related consequences among individuals with unhealthy alcohol use (defined as >=21 drinks/week or at least one episode/month with 6 or more drinks on the same occasion) and will prevent the increase of alcohol use among individuals without unhealthy alcohol use, compared to the control condition. Among individuals with unhealthy alcohol use, the study aims to detect a 3 drinks difference in the mean number of drinks/week at follow-up (1.5 drinks difference among individuals without unhealthy alcohol use) between subjects in the intervention and control groups (alpha level 0.05, 80% power).

**Methods:** Randomized controlled trial: 1080 men will be randomly assigned to receive electronic personalized feedback (n=600) or not (n=600). Participants recruited from the Swiss National Science Foundation supported Cohort study on Substance Use and Risk Factors (C-SURF) will be followed at 1 month and at 6 months. This will be a population-based sample of young French-speaking conscripts from a recruitment center. The primary outcomes will be weekly alcohol consumption and monthly frequency of risky single occasion drinking episodes, defined as 6 or more drinks per occasion. Analyses will be conducted separately for individuals with or without unhealthy alcohol use. The E-SBI will use a website which is structured in French, thus only French-speaking participants from C-SURF will be enrolled in this study.

**Expected value:** From an international perspective, this study will assess E-SBI efficacy in a population-based sample of young men, including potential primary prevention effects among moderate drinkers (who have not been studied extensively). From a national perspective, we know that face-to-face brief interventions conducted in the same setting are effective in decreasing alcohol use, but cannot be offered to everyone due to lack of resources. The proposed research will assess the efficacy of a procedure that could be implemented nationwide and include all conscripts.

KEYWORDS: unhealthy alcohol use, electronic screening and brief intervention, internet, primary and secondary prevention, young adults

# 2. Research plan

**2.1 Current state of research in the field**

***Screening and brief intervention for unhealthy alcohol use***

Unhealthy alcohol use (alcohol consumption that increases the risk of health consequences and includes abuse and dependence) is one of the leading causes of morbidity and mortality among young adults (1, 2). In Switzerland, there is high prevalence of risky single occasion drinking (also called binge drinking), especially among young men (3).

Key issue #1: unhealthy alcohol use is one of the leading causes of morbidity and mortality among young adults

There are major challenges related to unhealthy alcohol use. Most people with unhealthy alcohol use do not seek specialized treatment (4, 5), so efforts have been made to bring services (notably brief interventions) to them. Primary care physicians have been encouraged to provide screening and brief intervention (SBI), which has demonstrated success in reducing alcohol use within primary care settings (6-8), as well as among young individuals (9-11). In spite of the success of SBI delivered in primary care, there are several concerns regarding the feasibility of health care workers finding available resources for teaching and delivering SBI amidst competing priorities and tasks connected to routine medical practices and patient contact (12, 13). In addition, young individuals are unlikely to receive face-to-face SBI because they usually are not sick, and tend to have very limited contact with health care systems.

*Key issue #2: young individuals have limited contact with the health care system, thus are unlikely to receive face to face interventions for unhealthy alcohol use*

***Electronic screening and brief intervention***

As a result, the use of internet and computer-based electronic interventions has been increasing over the past years. It allows the delivery of brief interventions 24 hours a day at the subjects’ convenience in the comfort of their own homes, and gives access to a much broader population than that found in primary care facilities. It is also a relatively inexpensive procedure (14). Most of the known electronic screening and brief interventions (E-SBI) are based on social norms interventions, and contain personalized feedback. According to social norms theory, our perceptions and beliefs of what is “normal” behavior among others will influence our own behavior (15-18). Believing that others drink heavily will impact one’s own drinking. The difference between one’s actual behavior and what one thinks is true of others is considered a “misperception”, and research among college students has established that a high level of misperception is associated with higher levels of alcohol use, and that perceived norms are a strong predictor of alcohol use (18-20). Electronic and face-to-face SBI aiming for the reduction of this misperception (by means of normative feedback) are considered effective for college students (11). In addition to normative feedback, available E-SBIs offer a broad range of personalized feedback and information, such as feedback on blood alcohol concentration (BAC), the monetary impact of alcohol consumption, recommendations regarding drinking, suggestions on how to cut down or on how to avoid risky situations, etc.

[www.alcooquizz.ch](http://Www.alcooquizz.ch), an E-SBI website in French developed in Lausanne, Switzerland, was launched July 15 2010. From July 15 to August 15 2010, 13,427 individuals accessed the website, of whom 11,472 (85%) fully completed the assessment and received personalized feedback. The mean age (SD) was 36.6 (13.0), 68.6% were men, and 57.7% had an Alcohol Use Disorders Identification Test >8 (21, 22), indicating probable problem drinking. Men reported a mean of 15.3 drinks per week and women a mean of 9.0 drinks per week. E-SBI appears to be feasible in Switzerland, since more than half of those accessing the website had AUDIT scores >8.

Most of the published literature on E-SBI targeting alcohol use are results from North-America (USA and Canada) and Australia or New Zealand studies, conducted primarily among college student populations (23-28). Several studies have been conducted in the UK and Finland (29, 30). Other studies conducted in the general population indicate that E-SBI is also a valuable strategy within non-student populations (14, 31, 32). However, as reported in a 2009 Cochrane review (11), more evidence of efficacy for young individuals other than college students is needed.

Key issue #3: an electronic brief intervention is an opportunity to reach a wide population, but more evidence is needed on its efficacy among young individuals outside of college settings

Most published studies have focused on individuals with unhealthy alcohol use. E-SBIs, when made available for the general population, are likely to be used also by individuals with moderate drinking or abstinence. It is therefore important to know what impact these intervention can have on moderate drinkers or abstainers. Providing information on alcohol use and its consequences may help prevent possible increases in use among low-risk drinkers. Nevertheless, there is a lack of knowledge about the primary preventive effects of E-SBI. The use of E-SBI for primary prevention (i.e. future unhealthy alcohol use among individuals with moderate drinking or abstinence) and secondary prevention (i.e. decreasing alcohol use among individuals with unhealthy alcohol use, before the development of life threatening medical consequences) among a population-based sample of young men will give important information regarding its efficacy and feasibility. Young men are the most likely to benefit from reduction in alcohol use since they account for a high proportion of unhealthy alcohol users, with high rates of risky single occasion drinking (33).

**The proposed project will test the efficacy of an E-SBI for unhealthy alcohol use in a population sample of Swiss young men, using a primary and a secondary prevention approach.**

**2.2 Detailed research plan**

**2.2.1 Aims**

We propose to test the hypothesis that E-SBI targeting alcohol use delivered by the internet will improve drinking outcomes among young men. It is hypothesized that E-SBI will decrease later alcohol use and related consequences among individuals with unhealthy alcohol use and prevent any increase in alcohol use among individuals who do not engage in unhealthy use. In addition, since the proposed intervention offers access to many resources, such as information and fact sheets, we can determine the access and usage rates among young adults.

**2.2.2 Rationale**

Switzerland has a mandatory army recruitment process, thus presenting a unique opportunity to access the entire population at a given age. The impact of E-SBI in the general population can be evaluated, within a setting conducive to later implementations of the inventions. The proposed project will take advantage of an ongoing study supported by the Swiss National Science Foundation (SNF grant 33CSCO-122679): the Cohort study on Substance Use and Risk Factors (C-SURF) and recruit participants for the proposed project from among the subjects there. The E-SBI study will be a large nested project integrated with C-SURF.

French-speaking young men are currently recruited within the Lausanne army recruitment center. Since the recruitment procedure is mandatory (and C-SURF has no *a priori* exclusion criterion), virtually all French-speaking Swiss 19-year-old men are eligible for enrollment. In collaboration with the C-SURF team, participation in the present project will be offered to all French-speaking cohorts. This will allow E-SBI to be studied within a well-characterized population of young men drawn from the Swiss general population.

From a public health point of view, the young men in this population are of great interest since they frequently drink amounts of alcohol that put them at risk for damaging consequences (2, 3, 33). Preventing or decreasing unhealthy alcohol use in this population will have important public health implications. As stated earlier, since this population is mostly healthy and consequently do not usually access the health care system, new ways of delivering personalized interventions need to be developed. E-SBI represents a potentially effective option, since young men frequent the internet often. Therefore, this population is of particular interest for researching E-SBI.

From a national perspective, face-to-face brief interventions conducted at the recruitment center in Lausanne have proven to be effective in this population, showing a 20% reduction in weekly alcohol consumption six months after baseline (34). Because resources are not available for delivering face-to-face intervention to everyone attending recruitment centers, electronic alternatives represent an opportunity to extend this service to a wider base of potential recipients**.**

***Strengths:***

The Alcohol treatment center research group (N. Bertholet, J.-B. Daeppen, G. Gmel, M. Faouzi and J. Gaume) has been conducting studies at the army recruitment center in Lausanne since 2006 and has developed a strong research history within this setting, including a large randomized controlled trial of face-to-face brief intervention. The C-SURF project takes place in this setting. More so than other studies in the field, C-SURF collects extensive data; this furnishes detailed descriptions of all E-SBI participants. C-SURF procedures (e.g. the tracking team and up-to-date address registry) should help ensure low drop-out rates for the proposed project. Later on, if short or medium term effects of E-SBI are shown, the C-SURF follow-up will allow the evaluation of long term effects of E-SBI.

The Clinical Research Center (CRC) of the CHUV will be involved in the management and analysis of the data. This collaboration will help maintain a high level of methodological quality in the E-SBI component.

**2.2.3 Methods**

***Study design***

This will be a randomized, controlled clinical trial evaluating the impact of E-SBI on alcohol use. All participants who meet the eligibility criteria will be randomized into either of these groups; intervention or control. Screening and/or baseline evaluation will be done, with follow-ups at one month and at 6 months after randomization. The first follow-up will allow assessment of the relatively short-term result from the intervention. The one-month and the 6-month assessments will be done electronically. The study design is presented in Figure 1

# Figure 1: Study design

*Census of young French-speaking men*

*C-SURF participants (n=3500)*

Individuals invited to participate (n=3500)

No access to the website

or decline participation

Individuals completing the baseline assessment (n=1200)

Random allocation* (n=1200)

Control (n=600)

Intervention (n=600)

1 Month Electronic follow-up assessments

(n=960)

6 Month Electronic follow-up assessments

(n=900)

Alcohol consumption

* Participants will fill out the baseline assessment and then be immediately randomized. Participants assigned to the intervention group will receive the intervention at this time

# *Definition of the study population*

The study population will consist of 20-year-old, French-speaking Swiss men participating in the C-SURF cohort project. As stated before, C-SURF has no *a priori* exclusion criterion, so virtually all men showing up for the mandatory recruitment procedure will be eligible. The recruitment facility at Lausanne is the only center in the French-speaking sector of Switzerland, allowing a broad spectrum of young men (not limited to college students) to be reached. Since we propose adapting the website that currently is entirely in French, we will limit our inclusion to C-SURF participants who speak French (n=3500).

## *Inclusion criteria for the proposed project (no hierarchical order)*

1. Participants must be part of C-SURF
2. Participants must read French and provide informed consent.
3. Participants must be willing to be randomly assigned to an intervention or a control condition and agree to complete follow-up assessments.

## *Exclusion criteria (no hierarchical order)*

No specific exclusion criterion has been retained for the present study.

## *Efficacy measures*

### *Primary outcomes*

1. Weekly alcohol consumption (number of drinks per week, where one drink contains 10g of ethanol)
2. Monthly frequency of Risky Single Occasion Drinking episodes (RSOD corresponding to 6 or more drinks per occasion)

### *Secondary outcomes*

### Number of consequences due to alcohol (was injured or injured someone else, had a hangover, missed a class or work, performed poorly at work, did something that was later regretted, had a blackout, got into an argument or fight with friends, had unplanned sex, had unprotected sex, damaged property, had problems with the police, received medical treatment, observed negative impact on physical health, and observed negative impact on mental health)

### Computed Blood Alcohol Concentration (BAC), based on maximum reported alcohol consumption since previous assessment

### Presence of unhealthy alcohol use (>=21 drinks per week or at least one RSOD episode per month)

### Use of the website

***Study intervention***

The electronic intervention includes self-assessment of current alcohol use and consequences, personalized feedback on alcohol use, and general information on alcohol use and its consequences (i.e. “fact sheets”). E-SBI personalized feedback will be adapted from [www.alcooquizz.ch](http://www.alcooquizz.ch) and will consist of:

- Normative feedback (comparison of the participant’s alcohol consumption per week and per occasion to the consumption of individuals of the same age in the Swiss population, based on data from the Swiss Health Survey (35) with more than 12,000 participants).
- Feedback on reported consequences
- Calorific value of reported consumption
- Computed BAC based on maximum reported alcohol consumption, and potential consequences
- Indication of risk category (i.e. presence or absence of episodic risky drinking and/or weekly risky drinking, and probability of alcohol-related problems)

Participants who report unhealthy alcohol use will be encouraged to modify their drinking habits, and be presented with the rationale for risks associated with their current use of alcohol. They will be asked whether or not they intend to change their drinking habits, in order to enhance thoughts about change and to characterize participants in terms of motivation to change. According to motivational interviewing theory, encouraging people to envision hypothetical change helps them take steps towards that change (36). Participants will have the opportunity to print their personalized feedback form, and will be able to access a section containing general information on alcohol use and its consequences.

##

## *Control group*

Participants randomized into the control group will complete the same baseline assessment as do members of the intervention group, but will neither receive personalized feedback nor have access to the general information section on the website.

## *Time to complete the assessment*

The approximate time to complete the baseline assessment is three minutes. All participants will be asked if they think that the internet is a suitable option for providing personalized information about substance use.

### *Recruitment*

After giving informed consent to participate in C-SURF, subjects are told that they are likely to be offered participation in another study. Invitations will be sent via e-mail to C-SURF participants (most C-SURF procedures are already electronic) that contain a description of the E-SBI study and a unique identifier. Invitees will be instructed to go to the study website, enter their unique identifier and complete the on-line assessment if they agree to participate, or to indicate that they decline. In case of non-response, a maximum of two reminders will be sent. The website log linking subject addresses to their unique identifiers will be kept separate in order to preserve confidentiality.

### *Randomization*

Randomization will be processed (unseen) in the website background, using an automated randomization procedure that has been used in other studies in the field. Following the assessment, the web server software will randomly assign participants to either receive the intervention or not. This selection will be based on the order in which participants accessed the website and stratified by drinking status (unhealthy alcohol use yes vs no). None of the study personnel will be involved in the process, thus all allocations will be anonymous.

***Technical support***

A research associate will be hired to manage the transfers between the C-SURF database and the website coordinators, to contact non-responders, and to oversee and coordinate recruitment for the E-SBI study according to the C-SURF calendars and schedules. Study personnel will be available during business hours (and afterwards by e-mail) to answer all questions about using the website.

***Follow-up period***

Participants will be contacted by e-mail one week prior to each follow-up point. They will receive a unique identifier and will be asked to log on to the website and complete the follow-up assessment within one week. Those who do not complete the assessment will receive up to three e-mail reminders, followed by three additional reminders via telephone. This same procedure will be used at the 6-month follow-up. All follow-up assessments will be scheduled according to the initial date of randomization, which is considered Study Day 1.

###

### *Participant compensation*

Subjects will be offered compensation for their time and any inconvenience caused by their participation. They will receive 5 CHF vouchers as they complete the baseline, the first follow-up and the second follow-up steps. They can choose to purchase music online or donate the vouchers to a favorite charity.

**2.3.4 Data and data collection**

### *Instrument to evaluate the outcome*

The assessment at baseline and at each of the two follow-up points will consist of a self-completed electronic questionnaire that will include validated instruments and additional questions. Alcohol consumption will be collected via quantity/frequency questions (“In a given day that you use alcohol, how many drinks are you drinking?” / “On how many days in a regular week are you drinking alcohol?”). Additionally, participants will complete the Alcohol Use Disorders Identification Test (AUDIT)(21, 22). The AUDIT is a 10-question measure that has been extensively studied and validated, and has been used successfully in other electronic brief interventions (14, 27). Participants will also report their heaviest alcohol use on a single occasion over the past 3 months. Alcohol-related consequences will be assessed using 12 questions adapted from a questionnaire developed by Wechsler et al., 1994 (37). Subjects will be asked two supplemental questions about the impact of alcohol on their physical and mental health (“Over the past year, do you think your alcohol drinking had a negative impact on your physical health” / “your mental health?”).

Identical measures will be used at baseline and at each of the two follow-ups. When necessary, questions that are time-referenced (e.g. “over the past x months…”) will be adapted to accommodate each of the follow-up points (i.e. “over the past month…”, “over the past 5 months…”, or “since the last time we asked you about your drinking...”).

Statistics on website use (e.g. number of topics accessed, time spent on the feedback, etc.) will be collected for each participant.

***Sample size calculation***

We used data from a previous study conducted in the same setting and with the same population to approximate the alcohol consumption data (34), data from a recent publication in the field to approximate the response and attrition rates (28), and data from the [www.alcooquizz.ch](http://www.alcooquizz.ch) website to evaluate the proportion of individuals with unhealthy alcohol use.

Statistical considerations

The sample size calculations are based on the primary outcome variable comparing the mean number of drinks per week in the intervention group with the mean number of drinks per week in the control group. We have two different hypotheses based on whether or not participants have unhealthy alcohol use. In both cases, we will compare the mean number of drinks per week of the intervention group to those of the control group.

*Participants with unhealthy alcohol use:*

Null hypothesis: the mean number of drinks per week is the same between groups (alternative hypothesis: μ1≠μ2), mean number of drinks per week in the intervention group (SD): 15.0 (13.0), and in the control group (SD): 12.0 (13.0). Delta=3.0, with σ=13.0 in both groups. Considering 80% power to detect a 3 drinks per week difference in the mean number of drinks per week at follow-up (alpha level 0.05), we need 295 participants in each group to reject the null hypothesis.

*Participants without unhealthy alcohol use:*

Null hypothesis: the mean number of drinks per week is the same between groups (alternative hypothesis: μ1≠μ2), mean number of drinks per week in the intervention group (SD): 2.5 (4.7), and in the control group (SD): 4.0 (4.7). Delta=1.5, with σ=4.7 in both groups. Considering 80% power to detect a 1.5 drinks per week difference in the mean number of drinks per week at follow-up (alpha level 0.05), we need 155 participants in each group to reject the null hypothesis.

Based on previous studies in the same setting and on [www.alcooquizz.ch](http://www.alcooquizz.ch) data, we expect a >50% prevalence of unhealthy alcohol use among E-SBI participants (3). In each of the intervention and the control groups, we will need 295 participants with unhealthy alcohol use and 155 participants without unhealthy alcohol use (i.e. 450 participants per group).

According to results from other electronic interventions, we might expect the loss to follow-up to be 30% at 6 months. Using e-mail and telephone reminders and other C-SURF procedures, such as invitations and tracking teams, we should be able to reduce the loss to follow-up to 20% or less. The sample size (n=450 per group) was adjusted accordingly (n=600 per group). Previous studies in the field suggest that we should obtain a 50% refuse/decline rate among those accessing the website. In order to randomize 1200 participants into 600 participants per group, we will need to invite 2400 potential participants. Therefore, by inviting all C-SURF French-speaking participants (n=3500), we should be able to recruit an adequate number of subjects for the proposed project (Figure 1).

***Diagnostic statistics***

Descriptive statistics, such as mean, median, standard deviation, range, minimum, maximum, frequencies and plots, etc., will be computed for baseline, primary and secondary outcome variables. Except for the presence or absence of unhealthy alcohol use, primary and secondary outcomes are continuous. Randomization results, data distribution, variability and outliers on primary and secondary outcome data will all be checked. The diagnostic results will be used to describe the study population and to base statistical modeling and use of non-parametric tests. All exploratory analyses will be reported as such in publications.

***Efficacy analysis***

All analyses will be conducted separately for participants with and without unhealthy alcohol use in order to assess two specific hypotheses (i.e. decrease in subsequent alcohol use and related consequences among individuals with unhealthy alcohol use and no increase among individuals without unhealthy alcohol use). Efficacy analyses will be based on data collected at the E-SBI baseline, 1 month follow-up and 6 months follow-up.

*Primary efficacy analysis*

An intention-to-treat analysis will be used. In our primary analysis we will be testing the null hypothesis of no difference between mean number of drinks per week and number of RSOD episodes per month in the E-SBI and the control groups (μ1=μ2). For normally-distributed continuous data, t-tests for 2 independent samples will be calculated for the overall crude analysis. For data that are not normally-distributed, non-parametric Wilcoxon rank sum tests will be used. Then we will utilize an analysis of covariance model (ANCOVA) to control for covariates, which may include demographics, alcohol consumption, number of RSOD episodes per month and alcohol-related problems, all at baseline. Models of more complexity will take into account multiple observations, such as Generalized Estimating Equations (GEE), regressions or similar models.

*Secondary efficacy analysis*

Secondary continuous outcomes will be analyzed in the same manner as the primary outcomes.

*Additional analyses*

In addition to efficacy analyses, we will conduct usage analyses on specific website pages accessed by participants, including details of the time spent within each of the sections. These statistics will help us determine what information participants found most interesting, and assist us in further developing the electronic interventions.

***Confidentiality***

Individuals with unhealthy alcohol use are vulnerable because of the negative view that society holds toward unhealthy alcohol use and alcohol use disorders. This view may lead to prejudice and bias toward unhealthy alcohol users in professional, academic, or army environments, and make them prone to discrimination. Therefore, extra caution needs to be taken in research procedures to avoid prejudice towards those participants who report their own unhealthy alcohol use. This study will include all persons who access the website (not just those with unhealthy alcohol use), therefore, visiting the website per se should not present any risk of stigma.

All data will be kept strictly confidential. The names of participants, recognizable descriptors, and identification numbers will not be used in any publications. Each participant will receive a unique identifier for logging onto the website. The document linking these identifiers to personal data, such as names and e-mail addresses, will be kept in a separate location from other study material, and will not be accessible to anyone except the principal investigators**.** In addition, all screening will occur anonymously on the website only, and the results cannot be retrieved by anyone who is not part of the research team.

***Benefits and risks***

*Benefits*

There are several benefits from this study. The direct benefits include improvement in drinking outcome if E-SBI proves effective, and the assessments and other information that participants will receive as feedback might give them a heightened awareness of their drinking habits and encourage them to consume less alcohol. Although it is not a direct benefit, the monetary compensation offered to participants will help offset any additional costs that may be incurred because of the study.

*Risks*

The major risk in this research is breach of confidentiality, but as described earlier, great effort will be made to minimize that risk as much as possible. Electronic interventions have no reported or known side effects.

*Risk/benefit ratio*

The possible risk of harm to participants caused by this study is limited; therefore the potential benefits outweigh any potential risks.

**2.3 Schedule and milestones**

A research associate will be hired in July 2011. The research associate task will be to coordonate invitations to participate in collaboration with the C-SURF team and the company developing the website. The [www.alcooquizz.ch](http://www.alcooquizz.ch) website will be adapted to accommodate the follow-up sessions, to include various procedures added to the study, and to focus on targeting 20-year-old men. Reconstruction of the website will begin in April 2011. The completed version of the website and all electronic procedures will be ready for use in August 2011. At that time, invitations to participate in the E-SBI study will be sent to C-SURF participants. Invitations to participate will be sent by waves of 500 (one wave each week, September-October 2011). After inclusion and randomization in the E-SBI study, each participant will be contacted one month later for the first follow up, and six months later for the second follow-up. During the recruitment and follow-up phases, the research associate will verifiy on a daily basis the number of invited participants, the response rate, and data consistency and exhaustivity. Direct contacts will be made with the company developing the website on a weekly basis to assess enrollement and attrition, as well as to verify study procedures. During the follow-up phase, the research associate will contact subjects lost to follow-up by phone (after three reminders by e-mail), to encourage completion of the online questionnaire. The research associate will be employed at a 50% rate for the period of July 2011 to July 2012. The detailed schedule is presented in Figure 2.

**Figure 2: E-SBI study: schedule and milestones**

C-SURF participants

Census of young French-speaking men

Reconstruction of the website (adapted version of www.alcooquizz.ch)

MAY-AUGUST 2011

Individuals invited to participate

Individuals accessing the website

Random allocation

SEPTEMBER-OCTOBER 2011

MARCH-APRIL 2012

OCTOBER-NOVEMBER 2011

DATA CLEANING, ANALYSES, PRESENTATION OF RESULTS:

APR 2012 – DEC 2012

E-SBI STUDY

6 Month Electronic follow-up assessments

1 Month Electronic follow-up assessments

Control

Intervention

Alcohol consumption

C-SURF RECRUITMENT:

SEPT 2010-APRIL 2011

**2.4 Importance and impact**

On an international perspective, the proposed project intends to study E-SBI efficacy in a large sample of young men. Move evidence for the success of E-SBI is needed in this population. This research will add knowledge about the primary prevention impact of such intervention (i.e. among individuals without unhealthy alcohol use who may drink moderately). It will also yield important information about the feasibility and acceptability among young men of using this type of (electronic) intervention that is capable of furnishing feedback and information on alcohol use and its consequences privately and confidentially.

On a national perspective, the proposed project will help determine the efficacy of using an electronic means of targeting alcohol use among young men. Depending on its effectiveness, it could be a valuable alternative to face-to-face interventions (with known efficacy), and lead to a much broader implementation of E-SBI that focuses on alcohol consumption among Swiss conscripts. This could potentially have a significant and positive impact on public health.

***Interdisciplinary approach:***

The project will be conducted by investigators within institutions located in Lausanne and Toronto: The Alcohol treatment center (Département de médecine et santé communautaires, CHUV), the Clinical epidemiology center (Département universitaire de médecine et santé communautaires, CHUV and Lausanne University), The Center for Clinical Research (CHUV and Lausanne University), and the Center for Addiction and Mental Health (Toronto, Canada). This international collaboration will bring together an excellent group of researchers with specific expertise in all areas of the project.

We plan to publish the results of this study in peer-reviewed journals and to present research results at international meetings.

**REFERENCES**

1. Rehm J, Room R, Monteiro M, Gmel G, Graham K, Rehn N, et al. Alcohol as a risk factor for global burden of disease. Eur Addict Res. 2003;9(4):157-64.

2. Rehm J, Taylor B, Roerecke M, Patra J. Alcohol consumption and alcohol-attributable burden of disease in Switzerland, 2002. Int J Public Health. 2007;52(6):383-92.

3. Gmel G, Gaume J, Faouzi M, Kulling JP, Daeppen JB. Who drinks most of the total alcohol in young men--risky single occasion drinking as normative behaviour. Alcohol Alcohol. 2008;43(6):692-7.

4. Cunningham J, Breslin FC. Only one in three people with alcohol abuse or dependence ever seek treatment. Addict Behav. 2004;29:221-3.

5. Dawson DA, Grant BF, Stinson FS, Chou PS, Huang B, Ruan WJ. Recovery from DSM-IV alcohol dependence: United States, 2001-2002. Addiction. 2005;100(3):281-92.

6. Bertholet N, Schwan R, Duhamel O, Perney P, Daeppen JB. Efficacité de l'intervention brève. Alcoologie et Addictologie. 2003;25(45):59S-63S.

7. Bertholet N, Daeppen JB, Wietlisbach V, Fleming M, Burnand B. Reduction of alcohol consumption by brief alcohol intervention in primary care: systematic review and meta-analysis. Arch Intern Med. 2005;165(9):986-95.

8. Kaner EF, Beyer F, Dickinson HO, Pienaar E, Campbell F, Schlesinger C, et al. Effectiveness of brief alcohol interventions in primary care populations. Cochrane Database Syst Rev. 2007(2):CD004148.

9. Borsari B, Carey KB. Effects of a brief motivational intervention with college student drinkers. J Consult Clin Psychol. 2000;68(4):728-33.

10. Baer JS, Kivlahan DR, Blume AW, McKnight P, Marlatt GA. Brief intervention for heavy-drinking college students: 4-year follow-up and natural history. Am J Public Health. 2001;91(8):1310-6.

11. Moreira MT, Smith LA, Foxcroft D. Social norms interventions to reduce alcohol misuse in university or college students. Cochrane Database Syst Rev. 2009;3(CD006748).

12. Friedmann PD, McCullough D, Chin MH, Saitz R. Screening and intervention for alcohol problems. A national survey of primary care physicians and psychiatrists. Journal of general internal medicine. 2000;15(2):84-91.

13. Beich A, Gannik D, Malterud K. Screening and brief intervention for excessive alcohol use: qualitative interview study of the experiences of general practitioners. BMJ. 2002;325(7369):870.

14. Cunningham J, Wild TC, Cordingley J, van Mierlo T, Humphreys K. A randomized controlled trial of an internet-based brief intervention for alcohol abusers. Addiction. 2009;12(104):2023-32.

15. Berkowitz AD. An overview of the social norms approach. In: Lederman L, Stewart L, editors. Changing the culture of college drinking: A socially situated health communication campaign. Creskill, NJ: Hampton Press; 2005.

16. Perkins HW. Surveying the damage: a review of research on consequences of alcohol misuse in college populations. J Stud Alcohol Suppl. 2002(14):91-100.

17. Perkins HW. Misperception of peer drinking norms in Canada: Another look at the "reign of error" and its consequences among college students. Addict Behav. 2007;32(11):2645-56.

18. Perkins HW, Haines MP, Rice R. Misperceiving the college drinking norm and related problems: a nationwide study of exposure to prevention information, perceived norms and student alcohol misuse. J Stud Alcohol. 2005(66):470-8.

19. Kypri K, Langley JD. Perceived social norms and their relation to university student drinking. J Stud Alcohol. 2003;64:8.

20. McAlaney J, McMahon J. Normative beliefs, misperceptions, and heavy episodic drinking in a British student sample. J Stud Alcohol Drugs. 2007;68(3):385-92.

21. Saunders JB, Aasland OG, Babor TF, de la Fuente JR, Grant M. Development of the Alcohol Use Disorders Identification Test (AUDIT): WHO Collaborative Project on Early Detection of Persons with Harmful Alcohol Consumption--II. Addiction. 1993;88(6):791-804.

22. Gache P, Michaud P, Landry U, Accieto C, Arfaoui S, Wenger O, et al. The Alcohol Use Disorders Identification Test (AUDIT) as a screening tool for excessive drinking in primary care: reliability and validity of a French version. Alcohol Clin Exp Res. 2005;29(11):2001-7.

23. Copeland J, Martin G. Web-based interventions for substance use disorders: a qualitative review. J Subst Abuse Treat. 2004;26(2):109-16.

24. Kypri K, Saunders JB, Williams SM, McGee RO, Langley JD, Cashell-Smith ML, et al. Web-based screening and brief intervention for hazardous drinking: a double-blind randomized controlled trial. Addiction. 2004;99(11):1410-7.

25. Kypri K, Saunders JB, Gallagher SJ. Acceptability of various brief intervention approaches for hazardous drinking among university students. Alcohol Alcohol. 2003;38(6):626-8.

26. Kypri K, Sitharthan T, Cunningham JA, Kavanagh DJ, Dean JI. Innovative approaches to intervention for problem drinking. Curr Opin Psychiatry. 2005;18(3):229-34.

27. Saitz R, Palfai TP, Freedner N, Winter MR, Macdonald A, Lu J, et al. Screening and brief intervention online for college students: the ihealth study. Alcohol Alcohol. 2007;42(1):28-36.

28. Kypri K, Hallet J, Howat P, McManus A, Maycock B, Bowe S, et al. Randomized controlled trial of proactive web-based alcohol screening and brief intervention for university students. Arch Intern Med. 2009;169(16):1508-14.

29. Wallace P, Linke S, Murray E, McCambridge J, Thompson S. A randomized controlled trial of an interactive Web-based intervention for reducing alcohol consumption. J Telemed Telecare. 2006;12 Suppl 1:52-4.

30. Koski-Jannes A, Cunningham JA, Tolonen K, Bothas H. Internet-based self-assessment of drinking--3-month follow-up data. Addict Behav. 2007;32(3):533-42.

31. Saitz R, Helmuth ED, Aromaa SE, Guard A, Belanger M, Rosenbloom DL. Web-based screening and brief intervention for the spectrum of alcohol problems. Prev Med. 2004;39(5):969-75.

32. Linke S, Harrison R, Wallace P. A Web-based intervention used in general practice for people with excessive alcohol consumption. J Telemed Telecare. 2005;11 Suppl 1:39-41.

33. Rehm J, Taylor B, Room R. Global burden of disease from alcohol, illicit drugs and tobacco. Drug Alcohol Rev. 2006;25:503-13.

34. Daeppen JB, Bertholet N, Gaume J, Fortini C, Faouzi M, Gmel G. Efficacy of brief motivational intervention in reducing binge drinking in young men: A randomized controlled trial. Drug Alc Depend. 2010.

35. OFS. Enquête suisse sur la santé 2002. Neuchâtel: Office fédéral de la statistique, 2004.

36. Miller WR, Rollnick S. Motivational Interviewing: Preparing People for Change. 2nd ed. ed. New York, NY: The Guilford Press; 2002.

37. Wechsler H, Davenport A, Dowdall G, Moeykens B, Castillo S. Health and behavioral consequences of binge drinking in college. A national survey of students at 140 campuses. Jama. 1994;272(21):1672-7.
